# Broad Habitat Tolerance of the Alien *Duchesnea indica* (Andrews) Teschem. Across Urban Vegetation Communities in a Central European City

**DOI:** 10.3390/plants15192910

**Published:** 2026-09-23

**Authors:** Mirjana Šipek, Eva Horvat, Nina Šajna

**Affiliations:** Faculty of Natural Sciences and Mathematics, University of Maribor, Koroška Cesta 160, 2000 Maribor, Slovenia; eva.horvat3@student.um.si (E.H.); nina.sajna@um.si (N.Š.)

**Keywords:** invasive plant, urban flora, *Potentilla indica*, floristic homogenization, nutrients, micronutrients

## Abstract

*Duchesnea indica* (Andrews) Teschem. is an invasive alien species increasingly recorded in urban environments; however, its habitat characteristics, ecological preferences, and associated vegetation communities remain insufficiently studied in Central Europe. This study investigated the distribution and habitats of *D. indica* in the urban area of Maribor, Slovenia, aiming to assess its ecological amplitude across urban vegetation. Field surveys identified 47 sites, of which 24 vegetation plots were analyzed to characterize associated plant communities, while environmental conditions were assessed at 18 plots. The species was recorded in forest understories, forest edges, parking lots, lawns, and ruderal sites and showed the highest affinity for semi-shaded habitats. Habitats differed significantly in light availability, soil nitrogen and copper content, while soil moisture, pH, and other analyzed soil chemical properties did not differ significantly but displayed substantial variation within individual habitat. Cluster analysis revealed four vegetation clusters associated with *D. indica* habitats, which partially corresponded to the habitat groups defined along the light gradient. Ordination analysis further supported a partial differentiation of vegetation assemblages associated with contrasting light conditions, although the environmental variables did not explain a significant proportion of variation in species composition. These results indicate that *D. indica* occurs across a broad ecological range and can persist within heterogeneous urban vegetation communities. Its ability to establish under contrasting environmental conditions, particularly in disturbed and semi-shaded habitats, may contribute to its continued spread within urban landscapes and facilitate potential expansion into adjacent natural habitats.

## 1. Introduction

Urban environments comprise heterogeneous mosaics of semi-natural and anthropogenic habitats and often support a high proportion of alien plant species [1,2]. Frequent disturbances, intensive human activities, and the widespread cultivation of ornamental plants, many of which are of alien origin, facilitate the establishment and spread of non-native species [3]. Consequently, cities are increasingly recognized as important introduction points and reservoirs of alien species, acting as hotspots of alien plant diversity from which potentially invasive species may spread into surrounding semi-natural and natural ecosystems [4,5,6].

One such urban-associated alien plant species in Europe is *Duchesnea indica* (Andrews) Teschem. (syn. *Potentilla indica* (Andrews) Th. Wolf), commonly known as false strawberry, mock strawberry, or Indian strawberry. Native to Asia, this perennial species of the Rosaceae family has been introduced to all continents except Antarctica and has become naturalized in many regions worldwide. In Europe, it was introduced during the early nineteenth century as an ornamental plant cultivated in botanical gardens before escaping into the wild [7]. The earliest record of a naturalized population dates to Italy in 1869 [8]. Since then, the species has spread throughout much of Europe, including Germany, Austria, Switzerland, Poland, Slovakia, Croatia, Montenegro, and other countries [7,9,10,11,12,13,14]. For example, after being recorded as rare in Vienna in 1944, *D. indica* became widespread across the city within approximately five decades. By 2007, more than 220 localities had been documented across Germany, Austria, and Switzerland [10]. Today, *D. indica* is considered invasive in several European countries, as well as in North America and Australia [11].

Within its native range, *D. indica* occurs in mountain slopes, meadows, riverbanks, and other moist habitats up to 3100 m a.s.l. [15]. In its introduced range, it occupies a remarkably broad spectrum of habitats, ranging from shaded forest understories and forest edges [16] to open habitats such as lawns and meadows, as well as highly disturbed urban habitats including parking lots and roadsides [11,12]. In highly disturbed urban habitats, environmental conditions are modified by anthropogenic inputs, with elevated concentrations of nutrients and trace metals frequently resulting from traffic-related deposition, atmospheric pollution, and other urban processes. Although the species generally prefers moist and nutrient-rich habitats, its growth and performance are strongly influenced by disturbance and light availability [15,16]. Phenotypic plasticity and competitive ability under contrasting environmental conditions combined with stable ecophysiological performance [17,18,19,20,21] allows the species to persist under suboptimal conditions while rapidly exploiting newly available resources when environmental conditions improve [13,21].

In addition, vigorous clonal growth through creeping stolons that root at each node enables efficient local expansion, while its prolonged flowering period, extending from May to late October in the introduced range, increases opportunities for sexual reproduction and dispersal [22,23] and personal observations. The continued expansion of *D. indica* may be further facilitated by climate change. Liefländer and Lauerer [10] suggested that increasing temperatures could promote the further spread of the species throughout Central Europe. In Slovenia, *D. indica* is currently recorded predominantly in anthropogenically influenced habitats, where it often forms dense ground cover, although it is also observed in more natural habitats, particularly forest understories (personal observations).

In recent years, *D. indica* has attracted increasing scientific attention in Europe, reflecting its ongoing expansion and growing concern regarding its invasion potential [12,13,22,24]. Recent studies have primarily focused on phenotypic plasticity [17,19], competitive interactions [18,21], physiological integration [20], and the effects of selected environmental conditions on morphology and performance [13,21]. Field studies have also begun to characterize vegetation communities associated with *D. indica* in Central European cities [12,13]. However, despite numerous distribution records, detailed ecological characterization of occupied habitats and comprehensive assessments of the species’ ecological amplitude across contrasting environments remain scarce. In particular, the extent to which *D. indica* can tolerate environmental variation within heterogeneous urban landscapes remains poorly understood. Most existing records lack detailed habitat descriptions, and systematic evaluations of habitat characteristics are still limited. Addressing this knowledge gap is essential for understanding the mechanisms underlying invasion success of *D. indica*, predicting its future spread into semi-natural and natural ecosystems, and informing potential management strategies.

The aim of this study was to characterize the broad ecological range of urban habitats occupied by *D. indica* and identify environmental and floristic factors associated with its occurrence across contrasting urban habitat groups. Specifically, we aimed to: (1) determine the distribution of *D. indica* in the wider Maribor area and assess the range of urban habitats it occupies, from shaded forest understories to open habitats; (2) characterize the environmental conditions of *D. indica* habitats, with particular emphasis on photosynthetically active radiation, soil moisture, and soil chemical properties, including pH, nutrient concentrations, elemental composition (carbon, hydrogen, nitrogen and sulphur), and heavy metal content; and (3) compare the floristic composition of vegetation associated with *D. indica* among different habitat groups and evaluate whether the identified vegetation clusters correspond to habitat categories defined along a light availability gradient.

## 2. Results

### 2.1. Distribution and Habitat Occupancy of Duchesnea indica in Urban Area of Maribor

*Duchesnea indica* was recorded at 47 sites including forest edges, hedgerows, forest understories, parking lots, lawns, and other ruderal habitats within the urban area of Maribor (Figure 1). These habitats were subsequently classified into three habitat groups representing a light availability gradient: shaded habitats (e.g., forest understories), semi-shaded habitats (e.g., forest edges), and open habitats (e.g., grasslands and lawns).

*Duchesnea indica* exhibited the highest mean cover in shaded habitats (56.1%), whereas its mean cover was lower at semi-shaded and in open habitats. Across all 24 sampled plots, its cover ranged from 5% to 90% (Table 1). A total of 164 co-occurring plant species were recorded across the sampled *D. indica* habitats in urban area of Maribor.

The species most frequently recorded together with *D. indica* was *Geum urbanum* L., occurring in 83% of all plots (100% of semi-shaded plots, 86% of shaded plots, and 57% of open-habitat plots). Other common associated species included *Rubus fruticosus* L. agg. (46%; 50%, 43%, and 43%, respectively), *Urtica dioica* L. (42%; 70%, 14%, and 29%), *Oxalis fontana* Bunge (42%; 30%, 14%, and 86%), *Carpinus betulus* L. (42%; 50%, 57%, and 14%), and *Taraxacum officinale* F.H. Wigg. (42%; 50%, 14%, and 57%). *Sambucus nigra* L. was recorded in 38% of the plots occurring primarily in shaded forest understories (86%) and less frequently at semi-shaded habitats (30%). The occurrence of woody species in open habitats suggests that these sites are undergoing early successional changes.

### 2.2. Environmental Characteristics of Duchesnea indica Habitats

Habitat conditions varied considerably among the sampling sites, reflecting the broad ecological range occupied by *D. indica*. Photosynthetically active radiation (PAR) ranged from 12 to 1702 μmol m^−2^ s^−1^, with the lowest values recorded in shaded forest understories and the highest values in open habitats. Soil moisture varied between 6.7 and 33.8%, with the minimum recorded in open habitats and the maximum in shaded habitats. Habitat groups differed significantly in PAR (Kruskal–Wallis χ^2^ = 14.38, df = 2, *p* = 0.0008), whereas soil moisture did not differ among habitat groups (Kruskal–Wallis χ^2^ = 1.84, df = 2, *p* = 0.40). However, substantial variation in soil moisture was observed within habitat groups (Figure 2).

Soil pH ranged from 4.6 to 8.1, with the lowest values measured in shaded forest understories and the highest values in open habitats (Table 2). Soil organic carbon (C), nitrogen (N), and sulfur (S) contents ranged from 12 to 85 g kg^−1^, 0.7 to 5.8 g kg^−1^, and 0.1 to 1.2 g kg^−1^, respectively. Among the analyzed soil variables, only nitrogen content differed significantly among habitat groups, with higher concentrations in shaded habitats (Figure 3). Available potassium (K_2_O) and phosphorus (P_2_O_5_) ranged from 62.3 to 549.5 mg kg^−1^ and 74.2 to 289.6 mg kg^−1^, respectively (Figure 3; Table 2). The highest K_2_O concentration was recorded at an urban parking site overgrown at edges with trees (shaded habitat), whereas the lowest values occurred in open and semi-shaded habitats. Phosphorus (P_2_O_5_) exhibited a broadly similar pattern, although considerable variation was observed among individual sites within the same habitat group. Concentrations of trace elements also showed substantial variation among habitats (Table 2). Chromium (Cr) ranged from 61 to 162 mg kg^−1^, with mean concentrations of 87, 100, and 104 mg kg^−1^ in semi-shaded, shaded and open-habitat plots, respectively. Manganese (Mn) ranged from 0.49 to 1.1 g kg^−1^, with corresponding means of 0.81, 0.83, and 0.73 g kg^−1^. Iron (Fe) ranged from 26.8 to 44.2 g kg^−1^, with means of 36, 37, and 36 g kg^−1^, respectively. Nickel (Ni) ranged from 18 to 55 mg kg^−1^, with means of 38, 40, and 43 mg kg^−1^. Copper (Cu) ranged from 26.5 to 67.0 mg kg^−1^, with means of 35, 46, and 50 mg kg^−1^. Zinc (Zn) ranged from 58.5 to 215.5 mg kg^−1^, with means of 137, 169, and 155 mg kg^−1^. Arsenic (As) ranged from 7.5 to 25.5 mg kg^−1^, with means of 15, 16, and 17 mg kg^−1^, while lead (Pb) ranged from 13.5 to 70.5 mg kg^−1^, with means of 30, 44, and 38 mg kg^−1^, respectively. Overall, the wide ranges observed for both photosynthetically active radiation and soil properties indicate considerable environmental heterogeneity among habitats occupied by *D. indica*.

### 2.3. Vegetation Classification of Duchesnea indica Habitats

Hierarchical cluster analyses using Ward’s minimum variance (Ward.D2) and average linkage methods revealed a similar overall floristic structure among the 24 vegetation plots (Figure 4). The resulting clusters broadly corresponded to the predefined habitat groups, representing a gradient in light availability from shaded habitats like forest understories, through semi-shaded edge habitats and tree-overgrown parking areas, to open habitats (lawns and meadows). However, the separation among these habitat groups was not distinct. As all sampled plots were located within urban areas of Maribor or their immediate surroundings, many shared species associated with urban land use occurred across habitats, reducing floristic differentiation among them. This was primarily due to the widespread occurrence of ruderal species across all habitat groups and the presence of woody species even in open habitats as could be seen in chapter 2.1, reflecting ongoing vegetation succession. Nevertheless, both clustering methods showed a tendency for plots from shaded habitats to group together and for plots from open habitats to form separate clusters, whereas semi-shaded (edge) habitats generally occupied an intermediate position.

Detrended Correspondence Analysis (DCA) indicated that the length of the first ordination axis was 4.49 standard deviation (SD) units, suggesting a relatively long ecological gradient and a unimodal species response (Figure 5). Consequently, Canonical Correspondence Analysis (CCA) was used to examine the relationships between vegetation composition and the measured environmental variables.

The ordination scores of *D. indica* (DCA1 = 0.228, DCA2 = −0.059) placed the species close to the centre of the ordination space as expected, indicating that it is not strongly associated with any single vegetation group but occurs across a broad range of habitat conditions. To facilitate interpretation, *D. indica* was manually labelled in the DCA and CCA diagram while retaining its original ordination coordinates (Figure 5 and Figure 6).

Canonical Correspondence Analysis (CCA) was used to examine the relationship between vegetation composition and ecological variables representing light availability and soil moisture. Permutation tests indicated that the overall CCA model was not statistically significant (ANOVA, *p* = 0.42). Likewise, neither light (*p* = 0.12) nor soil moisture (*p* = 0.88) explained a significant proportion of the variation in species composition. These results suggest that differences in vegetation composition among the sampled *D. indica* habitats cannot be explained solely by the measured environmental variables and that additional abiotic or biotic factors likely contribute to the observed patterns.

Although the CCA model was not statistically significant, the ordination suggested differences in species composition among the three habitat groups representing a light availability gradient (open, semi-shaded, and shaded habitats; Figure 6). Plots from open habitats tended to be separated from those of shaded and semi-shaded habitats, whereas the latter two groups showed considerable overlap. The ordination also suggested that shaded habitats were characterized by a more distinct species assemblage, while species composition in open habitats was more variable. Considerable overlap among habitat groups was expected because urban land use promotes the occurrence of numerous ruderal and generalist species across different habitats.

In the ordination space, plots from open habitats were associated with higher light availability, whereas those from shaded and semi-shaded habitats were associated with relatively higher soil moisture. No clear relationship was observed between vegetation composition and the measured soil variables. Although soil nitrogen content differed significantly among habitat groups, soil variables were excluded from the final CCA because preliminary analyses indicated that they did not explain variation in vegetation composition. Soil variables showed an almost complete lack of association (*p* ≈ 1), as described in the Section 4 (Materials and Methods).

## 3. Discussion

Our results confirm that *D. indica* is a habitat-generalist species rather than restricted to a particular vegetation type or environmental setting, supporting recent observations from Central Europe [13]. By quantifying the ecological characteristics of habitats occupied by *D. indica* across the wider Maribor area, we demonstrated that the species possesses a broad ecological amplitude, enabling it to establish throughout a wide range of urban habitats along the urban–rural gradient. The species occurred in closed-canopy forest understories, forest edges, hedgerows, lawns, grasslands, and ruderal habitats, illustrating its ability to persist under contrasting environmental conditions. Although *D. indica* has traditionally been associated with anthropogenically disturbed habitats owing to its ornamental origin, gardens have most likely served as the initial source of introduction, after which the species has progressively expanded into surrounding habitats [3]. *Duchesnea indica* tolerates anthropogenic disturbances but can also persist in relatively stable semi-natural habitats. Its occurrence in frequently managed habitats, such as lawns, may be facilitated by its low-growing creeping growth form, which allows it to avoid severe biomass losses caused by mowing compared with taller competing species. This combination of disturbance tolerance and persistence across less disturbed habitats represents an important trait combination for successful urban invaders. Our field observations indicate that expansion of *D. indica* is no longer confined to urban environments but increasingly includes semi-natural habitats, particularly forest edges and understories adjacent to urban areas [16,25]. As propagule pressure continues and suitable habitats remain available, further spread into forests located farther from urban centers appears likely.

The three habitat groups as identified in this study differed primarily in light availability, expressed as photosynthetically active radiation. Nevertheless, *D. indica* occurred throughout the entire light gradient, from deeply shaded forest understories to fully exposed open habitats. Interestingly, field observations revealed that the species attained its highest cover in shaded habitats despite similar total vegetation cover among habitat groups. This pattern differs from controlled experimental studies, in which *D. indica* produced significantly greater biomass under higher light availability, particularly when nutrient availability was also elevated [21]. Such contrasting responses suggest that field performance is influenced not only by resource availability but also by interactions with coexisting species. In our study, plant species richness was the lowest in forest understories, where reduced interspecific competition may facilitate greater local dominance of *D. indica*. Consequently, the realized abundance of the species in natural communities is likely determined by the combined effects of abiotic conditions and biotic interactions rather than by light availability alone.

The environmental heterogeneity of *D. indica* habitats was also reflected in the pronounced variation in soil chemical properties. Urban soils are inherently heterogeneous owing to differences in land use, vegetation cover, management practices, atmospheric deposition, and historical disturbances [26,27,28]. Consequently, substantial spatial variation in soil pH, nutrient availability, organic matter, and elemental composition is expected even over relatively short distances.

Soil pH in *D. indica* habitats ranged from moderately acidic to moderately alkaline (4.6–8.1), with a mean value of 6.9, corresponding to predominantly neutral soils where the availability of both macro- and micronutrients is generally favourable for plant growth [29]. Nevertheless, *D. indica* also occurred at sites with considerably more acidic or alkaline soils. At lower pH values, the availability of several macronutrients may decrease because of fixation with metal ions, whereas alkaline conditions can reduce the availability of micronutrients [29]. The occurrence of *D. indica* across this wide pH gradient indicates that soil reaction alone is unlikely to limit its establishment within urban habitats.

Among the measured nutrients, nitrogen was the only variable that differed significantly among habitat groups, with the highest concentrations recorded in shaded habitats. This pattern is likely associated with greater organic matter accumulation beneath closed tree canopies and slower decomposition rates, which promote nitrogen retention in forest soils. Nevertheless, *D. indica* occurred across a remarkably broad nitrogen gradient, from 0.7 to 5.8 g kg^−1^. Phosphorus and potassium concentrations likewise indicated relatively fertile soils, particularly compared with many natural ecosystems [30]. Such nutrient-rich conditions are characteristic of urban soils, where fertilization, organic matter accumulation, and other anthropogenic activities frequently enhance nutrient availability [26,31].

The carbon-to-nitrogen ratio provides additional insight into the quality of soil organic matter and nutrient cycling. The average C:N ratio of approximately 17 in *D. indica* habitats exceeded the reported European topsoil average of 13.2 [30], although considerable spatial variability is expected in urban soils because anthropogenic disturbances modify both biogeochemical cycling and physical soil properties [27]. Higher C:N ratios are generally associated with slower decomposition and reduced nitrogen mineralization, as commonly observed in oligotrophic ecosystems such as coniferous forests and peatlands, whereas lower ratios typically characterize more nutrient-rich soils with greater nitrogen availability [30]. The relatively high carbon content (approximately 50 g kg^−1^) further indicates substantial organic matter accumulation in many of the sampled habitats. In experimental studies both Littschwager et al. [18] and Kreća et al. [21] demonstrated that *D. indica* responds positively to increased resource availability (e.g., nutrients and light) by increasing biomass production. Littschwager et al. [18] reported that *D. indica* achieved greater competitive success than the native *Fragaria vesca* L. through preferential allocation to aboveground biomass, enabling more effective light capture and competitive growth. In contrast, Kreća et al. [21] found that the ecologically similar native *Glechoma hederacea* L. produced greater biomass than *D. indica* under experimental conditions. These contrasting findings illustrate that competitive outcomes are strongly context dependent and vary among competitor species. Our field observations support this conclusion, as *D. indica*, *G. hederacea*, and *F. vesca* coexist within the same urban habitats in 29% and 17% of sampled sites, respectively, indicating that community composition is determined by complex interactions among resource availability, competition, disturbance, and other habitat characteristics. The frequent co-occurrence of *G. hederacea* is particularly notable, as this native clonal species shares several ecological characteristics with *D. indica*, including a creeping growth form, tolerance of disturbance, and the ability to persist under variable environmental conditions [21,32].

Consistent with the variation observed in nutrient availability, concentrations of essential micronutrients and potentially toxic trace elements also varied considerably among and within habitat groups. Iron was the dominant metallic element, with concentrations typical of mineral soils and largely determined by the geological parent material [33]. In contrast, copper, zinc, lead, and arsenic exhibited greater variability, consistent with the well-documented enrichment of urban soils through traffic emissions, building materials, industrial activities, and other urban sources [26,34]. Although concentrations of several trace elements were elevated at some sites, they remained within ranges commonly reported for urban soils and below levels generally considered phytotoxic for many plant species [33]. Williams et al. [35] further demonstrated that urban floras are typically composed of species capable of tolerating wide variation in nutrient availability, alkalinity, and other environmental conditions. The ability of *D. indica* to persist across this broad chemical gradient is therefore consistent with the suite of traits commonly associated with successful urban plant species.

The analysis of vegetation composition revealed that *D. indica* is not restricted to a single plant community but occurs across a broad spectrum of urban vegetation types, ranging from forests and forest-edge communities to meadows and ruderal habitats. Nevertheless, its associated species composition indicates a strong relationship with disturbed, nutrient-rich, and moderately moist vegetation. The most frequently accompanying species, including *Geum urbanum*, *Urtica dioica*, *Galium aparine*, *Aegopodium podagraria*, and *G. hederacea*, are characteristic of nitrophilous forest-edge and ruderal communities, particularly alliances *Aegopodion podagrariae* Tüxen 1967 and *Geo urbani-Alliarion officinalis* Lohmeyer et Oberd. in Görs et Müller 1969. These communities are commonly associated with increased nutrient availability, anthropogenic disturbance, and transitional habitats between semi-natural and urban environments.

Although habitat groups differed significantly in light availability, vegetation composition showed only weak differentiation among habitats. This indicates that urbanization may override natural environmental gradients by promoting the establishment of generalist synanthropic species and increasing the presence of alien plants, resulting in floristic homogenization within urban areas [36,37]. Similar homogenizing effects have been observed in cities where anthropogenic disturbance reduces differences between traditionally distinct habitat types and allows ruderal and invasive species to penetrate multiple vegetation communities. Consequently, *D. indica* appears to function as a species capable of linking otherwise distinct urban vegetation types and contribute to homogenizing effect.

The broad habitat spectrum occupied by *D. indica* reflects one of the characteristics commonly associated with successful invasive species [32] and recognition that successful invasive plants are not restricted only to resource-rich environments. Funk and Vitousek [38] demonstrated that many invasive species exhibit high resource-use efficiency, allowing them to maintain growth even under limiting light, water, or nutrient availability. Such physiological plasticity may partly explain the ability of *D. indica* to occupy habitats spanning wide environmental gradients despite pronounced differences in soil fertility and light availability. Rather than simply tracking favourable environmental conditions, the species appears capable of exploiting heterogeneous resource availability through plastic growth responses, physical integration between daughter ramets [20] and efficient resource use [18,21]. *Duchesnea indica* invasive success is probably a combination of physical integration, clonal growth, and tolerance to heterogeneous environmental conditions.

Physical integration may be particularly advantageous in urban environments, where habitats are spatially heterogeneous and frequently disturbed. For example, experimental studies have shown that *D. indica* can benefit from environmental heterogeneity through clonal integration, with connected ramets achieving higher performance under heterogeneous saline conditions [20]. This mechanism may also contribute to its success in urban habitats, where de-icing salt application during winter can create localized increases in soil salinity along roads and other disturbed areas. Although it was frequently associated with nutrient-rich communities, the lack of a clear relationship between vegetation composition and measured environmental variables indicates that it does not simply track highly productive habitats. Instead, its distribution across a broad nutrient and light gradient suggests that biotic interactions, propagule pressure, habitat history, and disturbance regime may represent additional important filters determining its establishment.

Disturbance is an important and well-recognized factor promoting the abundance and diversity of alien plants [39]. Species-level disturbance indicator values and the resulting disturbance index, which integrates the severity and frequency of disturbances associated with different habitats [40], could therefore provide additional insights into the vegetation dynamics underlying the occurrence and abundance of *D. indica* along an urban–rural gradient. However, we did not include such an index in the present study because this would extend the scope beyond our primary objectives, which were to characterize the habitat breadth of *D. indica* within an urban environment and to evaluate its occurrence across different vegetation communities and environmental conditions. Instead, we focused on directly measured environmental variables and vegetation composition rather than on developing a composite measure of anthropogenic and natural disturbance. Furthermore, all sampling sites were located within the urban area of Maribor and therefore shared a common anthropogenic context, although the intensity and type of management and disturbance undoubtedly varied among sites. A broader sampling design extending beyond the urban area would be needed to capture a more pronounced urban–rural disturbance gradient. Explicitly quantifying disturbance along such a gradient and evaluating its relative contribution to the occurrence and abundance of *D. indica*, would therefore represent a valuable direction for future research.

## 4. Materials and Methods

### 4.1. Study Site

The study was conducted in the urban area of Maribor, northeastern Slovenia. The region has a temperate continental climate with moderately warm summers and relatively cold winters. According to the climatological normals for the period 1991–2020, the mean annual temperature in Maribor is 10.2 °C, with mean monthly temperatures ranging from −0.1 °C in January to 20.3 °C in July. The mean annual precipitation is 949 mm, with precipitation occurring throughout the year and a slight maximum during the summer months. The meteorological data were obtained from the Slovenian Environment Agency for the Maribor meteorological station [41].

### 4.2. Vegetation Surveys and Environmental Measurements

Twenty-four vegetation plots (relevés) at sites occupied by *D. indica* was sampled during June and July 2020 (DI1–DI24; Table A1). Vegetation surveys followed the standard Central European phytosociological method of Braun–Blanquet [42]. Sampled plots were classified into three habitat groups (shaded, semi-shaded, open) based on light availability. The size of vegetation plots varied depending on the extent of homogeneous vegetation patches; however, all plots remained within the recommended size ranges for the respective vegetation types according to the Braun–Blanquet approach. The exact size of each relevé is provided in Table A1 in Appendix A. Plant nomenclature followed Martinčič et al. [43].

Among 24 vegetation plots 18 were selected for determination of environmental factors and soil characteristics (Figure 1). These variables were measured at three randomly selected points, approximately evenly spaced within each plot. Air temperature was measured using a digital thermometer (HI 98501; Hanna Instruments, Woonsocket, RI, USA), photosynthetically active radiation (PAR) was measured using a quantum meter (Apogee Instruments, Logan, UT, USA), and soil moisture was measured using an HH2 moisture meter (Delta-T Devices Ltd., Cambridge, UK). Soil samples were also collected from three randomly selected points using a soil corer with a diameter of 5 cm to a depth of 7.5 cm. The three subsamples collected within each plot were pooled into a single composite sample, air-dried, and ground prior to chemical analyses. Soil pH was determined potentiometrically in a 1:5 soil:water suspension and in 1 M KCl, using a combined glass electrode, according to ISO 10390:2005 (Soil quality—Determination of pH. International Organization for Standardization: Geneva, Switzerland, 2005). Elemental composition (C, H, N and S) were determined following standard laboratory procedures by the dry combustion method using an elemental analyzer (vario MACRO cube; Elementar Analysensysteme GmbH, Langenselbold, Germany). Physiologically available phosphorus and potassium (P_2_O_5_ and K_2_O, respectively) were extracted using the Egner–Riehm–Domingo lactate-acetate method [44]. Phosphorus was determined by spectrophotometry, while potassium was determined by flame spectrometry. Metals (Cr, Mn, Fe, Ni, Cu, Zn, As, Pb) were determined using a portable X-ray fluorescence (pXRF) analyser, specifically the Vanta handheld XRF analyser C Series (Olympus Corporation, Tokyo, Japan).

### 4.3. Data Analysis

#### 4.3.1. Vegetation Classification

Hierarchical agglomerative cluster analysis was used to classify the 24 vegetation plots (Figure 1) containing *D. indica* into distinct vegetation groups. Species abundance data, recorded using the Braun–Blanquet cover-abundance scale, were converted to percentage cover using the midpoints of the corresponding cover classes [42]. The resulting species-by-site matrix comprised 165 plant species and was highly sparse, with approximately 95% of all matrix entries equal to zero, a characteristic typical of vegetation datasets. Such data are well suited to analysis using the Bray–Curtis dissimilarity measure, which is robust to sparse community data and unaffected by joint absences [45]. Pairwise floristic dissimilarities among plots were calculated using the Bray–Curtis distance (vegdist() function, vegan package) [46] in R (version 2.5-7). An initial hierarchical cluster analysis was performed using Ward’s minimum variance method (Ward.D2 algorithm; hclust() function, stats package) to evaluate the overall structure of the vegetation data and identify the most meaningful partitioning of the plots. Subsequently, percentage cover values were square-root transformed to reduce the influence of dominant species while retaining information from less abundant taxa. A second hierarchical cluster analysis was performed using the unweighted pair-group method with arithmetic mean (average linkage). The vegetation classifications were conducted as an exploratory analysis to identify patterns in the vegetation composition and to determine whether the plots could be grouped according to their floristic similarity. In contrast, the three habitat groups were defined based on the light conditions of the sampling plots and represented the main environmental gradient considered in our study used in the subsequent analyses.

For visualization, dendrogram labels were coloured according to habitat group to facilitate interpretation of the relationship between floristic composition and habitat affiliation.

#### 4.3.2. Ordination of Vegetation Composition

Species composition was further explored using Detrended Correspondence Analysis (DCA) to assess the length of the underlying ecological gradient and determine the appropriate constrained ordination method. Because the length of the first DCA axis exceeded 4 standard deviation (SD) units, indicating a unimodal species response along the environmental gradient, Canonical Correspondence Analysis (CCA) was selected to investigate the relationships between vegetation composition and measured environmental variables. CCA was initially performed using light availability (PAR), soil moisture, and soil nitrogen content as explanatory variables. Following model simplification, soil nitrogen was excluded from the final model because it had no significant explanatory effect on vegetation composition (*p* ≈ 1). The significance of the overall CCA model, individual environmental variables, and canonical axes was evaluated using Monte Carlo permutation tests (999 permutations).

Species composition was further explored using Detrended Correspondence Analysis (DCA) to assess the length of the underlying ecological gradient and determine the appropriate constrained ordination method. Because the length of the first DCA axis exceeded 4 standard deviation (SD) units, indicating a unimodal species response along the environmental gradient, Canonical Correspondence Analysis (CCA) was selected to investigate the relationships between vegetation composition and measured environmental variables [47]. CCA was performed using light availability and soil moisture as explanatory environmental variables. The ordination results were visualized by plotting vegetation plots in reduced-dimensional space, with plots coloured according to the habitat group. This approach allowed assessment of the degree of separation and overlap among the identified vegetation units and predetermined habitat groups in relation to their floristic composition. The significance of the overall CCA model, individual environmental variables, and canonical axes was evaluated using Monte Carlo permutation tests (999 permutations). All ordination analyses were conducted in R (version 2.5-7) using the vegan package [46].

#### 4.3.3. Comparison of Environmental Conditions Among Habitats

Each habitat group was described based on the most frequently occurring species associated with *D. indica*, allowing identification of characteristic species assemblages and differences in species composition among habitat groups. In addition, environmental variables (light availability, soil moisture) were compared among habitat groups to evaluate whether floristic differences were associated with contrasting habitat conditions.

Prior to statistical analyses, the assumptions of normality and homogeneity of variances were evaluated for light and moisture measurements within each habitat group using the Shapiro–Wilk test and Levene’s test, respectively. Depending on whether these assumptions were met, differences among habitat groups were assessed using either one-way analysis of variance (ANOVA) followed by Tukey’s honestly significant difference (HSD) post hoc test or the non-parametric Kruskal–Wallis test followed by Dunn’s post hoc test. Because the assumptions of normality and/or homogeneity of variances were not satisfied, differences among habitat groups were analysed using the Kruskal–Wallis test with Dunn’s post hoc test and Benjamini–Hochberg correction for multiple comparisons. The distributions of light and moisture measurements across habitat groups were visualized using boxplots. All statistical analyses were conducted in R version 4.5.2 [48].

## 5. Conclusions

*Duchesnea indica* is a widespread species in the Maribor urban area, occurring across a broad range of urban habitats. It shows the highest frequency in semi-shaded and moderately disturbed environments, while the highest cover has in shaded habitats. The combination of vegetation analyses and distribution records demonstrates its strong association with anthropogenic habitats, while its presence along forest edges and in understory indicates its capacity to expand into less disturbed and more natural vegetation types. The absence of significant differences in soil moisture, pH, and most measured soil chemical properties among three habitat groups, together with substantial within-habitat variation, suggests that *D. indica* tolerates a broad range of soil conditions and can persist in chemically heterogeneous urban soils. Our results indicate that light availability and soil chemistry alone is unlikely to represent the primary environmental filter determining its occurrence. Instead, the successful establishment of *D. indica* appears to be driven by a combination of efficient resource acquisition, disturbance tolerance, and clonal growth, enabling it to exploit the environmental heterogeneity characteristic of urban ecosystems. These traits likely contribute to its persistence and continued spread within urban landscapes. Further long-term monitoring is therefore recommended to evaluate its expansion dynamics and assess potential impacts on native plant communities.

## Figures and Tables

**Figure 1:**
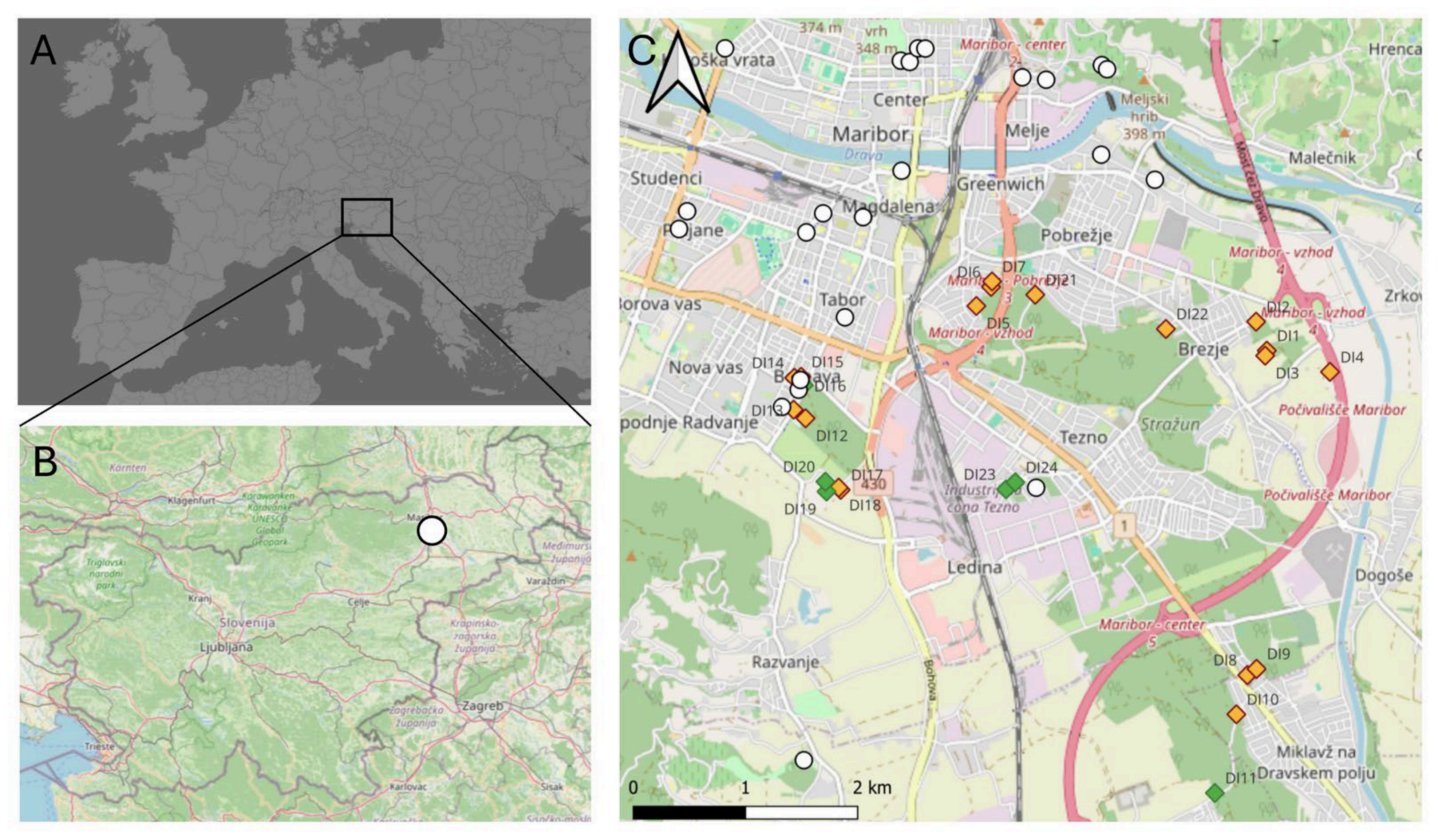
Study area and sampling locations. (**A**) Map of Europe showing the location of Slovenia (boxed). (**B**) Map of Slovenia indicating the city of Maribor (circled), where the study was conducted. (**C**) Distribution of *Duchesnea indica* populations in Maribor. White circles indicate recorded occurrences of *D. indica*. Diamonds represent sampling sites (*n* = 24); green diamonds indicate locations where only vegetation data were collected, whereas orange diamonds indicate locations where both vegetation and environmental data were collected.

**Figure 2:**
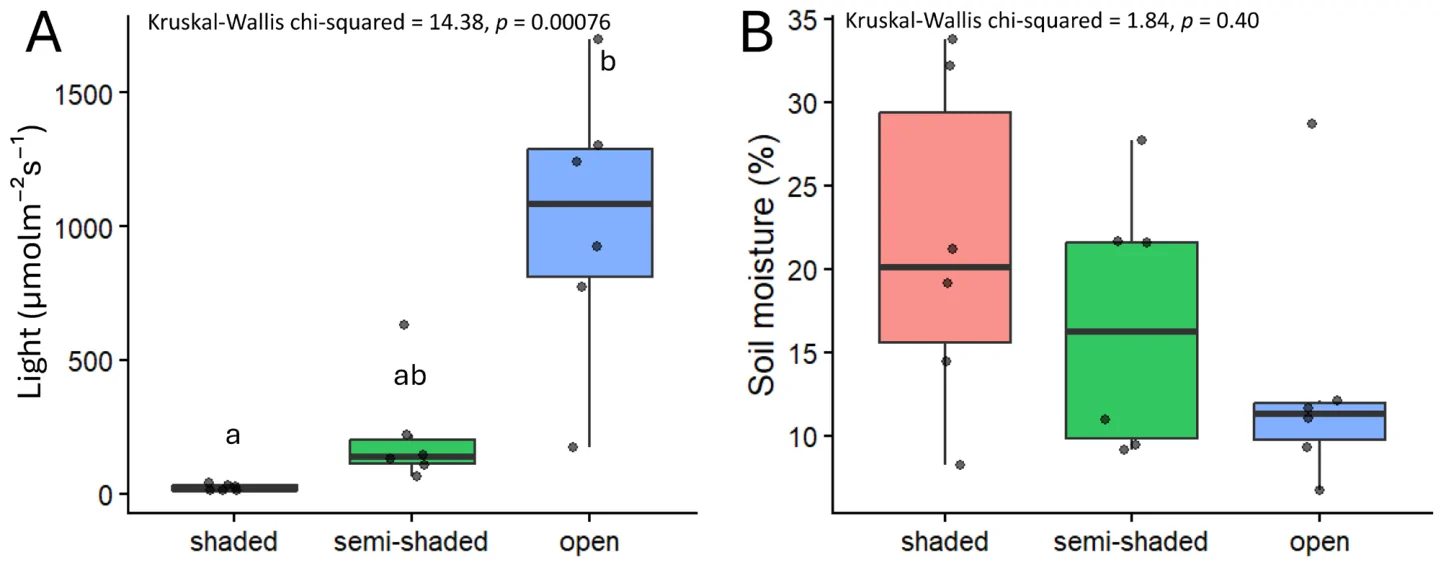
Differences in environmental conditions among *Duchesnea indica* urban habitat groups. (**A**) Photosynthetically active radiation and (**B**) soil moisture gradient across the three habitat groups (shaded, semi-shaded, and open habitat). Boxes represent the interquartile range (IQR), the horizontal line within each box indicates the median, whiskers extend to 1.5 × IQR, and points represent individual sampling sites. Different letters indicate significant differences among habitat groups according to Dunn’s post hoc test (*p* < 0.05).

**Figure 3:**
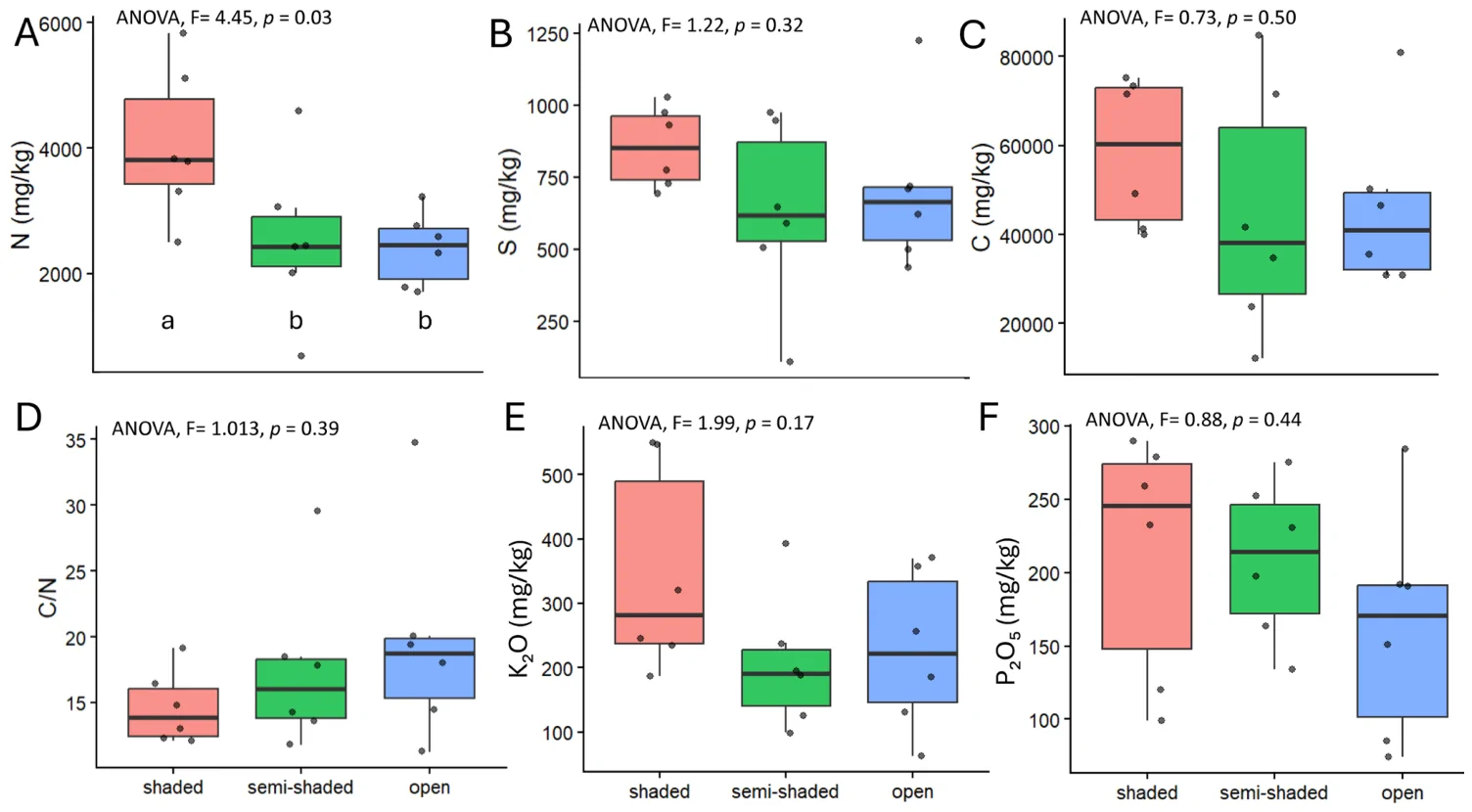
Soil variables characterizing nutrient status across the three *Duchesnea indica* habitat groups: (**A**) nitrogen (N), (**B**) sulfur (S), (**C**) carbon (C), (**D**) carbon-to-nitrogen ratio (C/N), (**E**) potassium oxide (K_2_O), and (**F**) phosphorus pentoxide (P_2_O_5_). Boxplots show the median, interquartile range, whiskers (1.5 × IQR), and outliers. Different letters indicate significant differences among habitat groups according to Tukey’s HSD test (*p* < 0.05).

**Figure 4:**
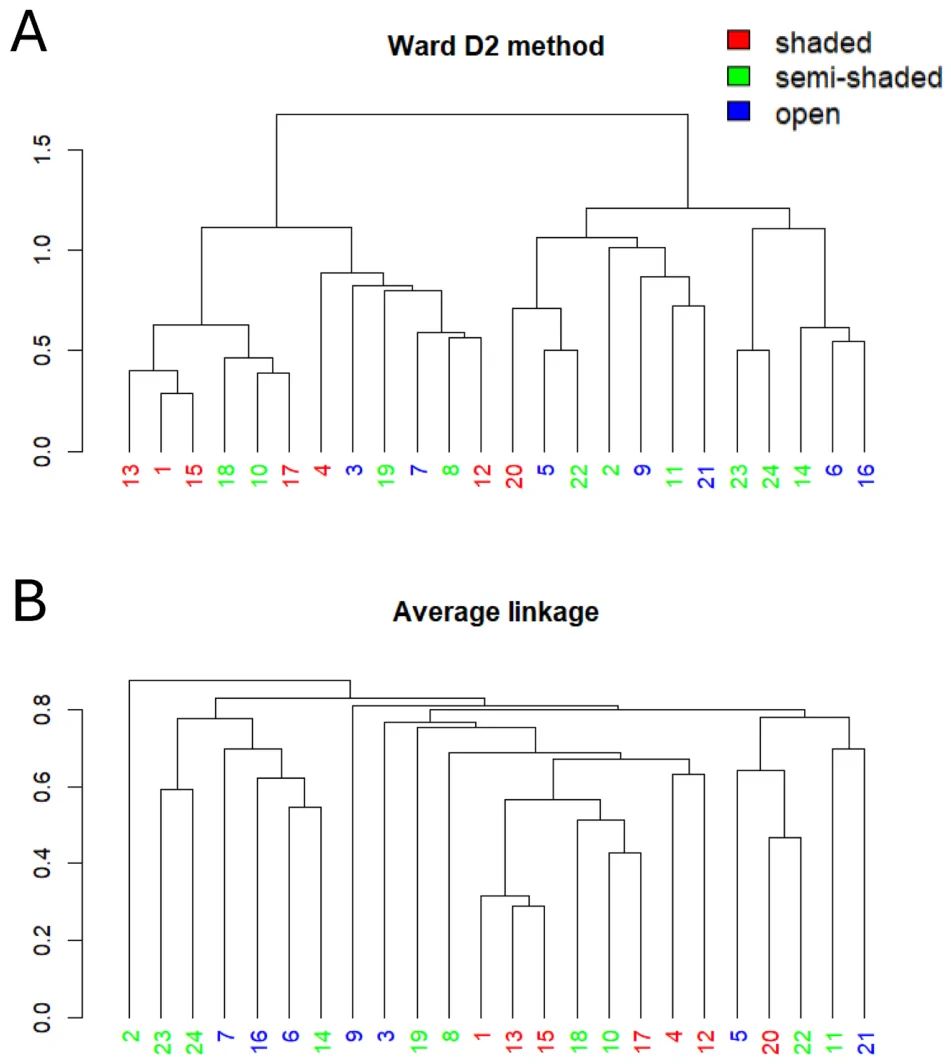
Hierarchical cluster analysis of the 24 vegetation plots containing *Duchesnea indica* based on Bray–Curtis dissimilarity. (**A**) Dendrogram obtained using Ward’s minimum variance method (Ward.D2), illustrating the overall floristic structure and supporting the identification of vegetation groups. (**B**) Dendrogram obtained using the unweighted pair-group method with arithmetic mean (average linkage) based on square-root transformed percentage cover data, showing the final classification of vegetation groups. Plot labels are coloured according to habitat group.

**Figure 5:**
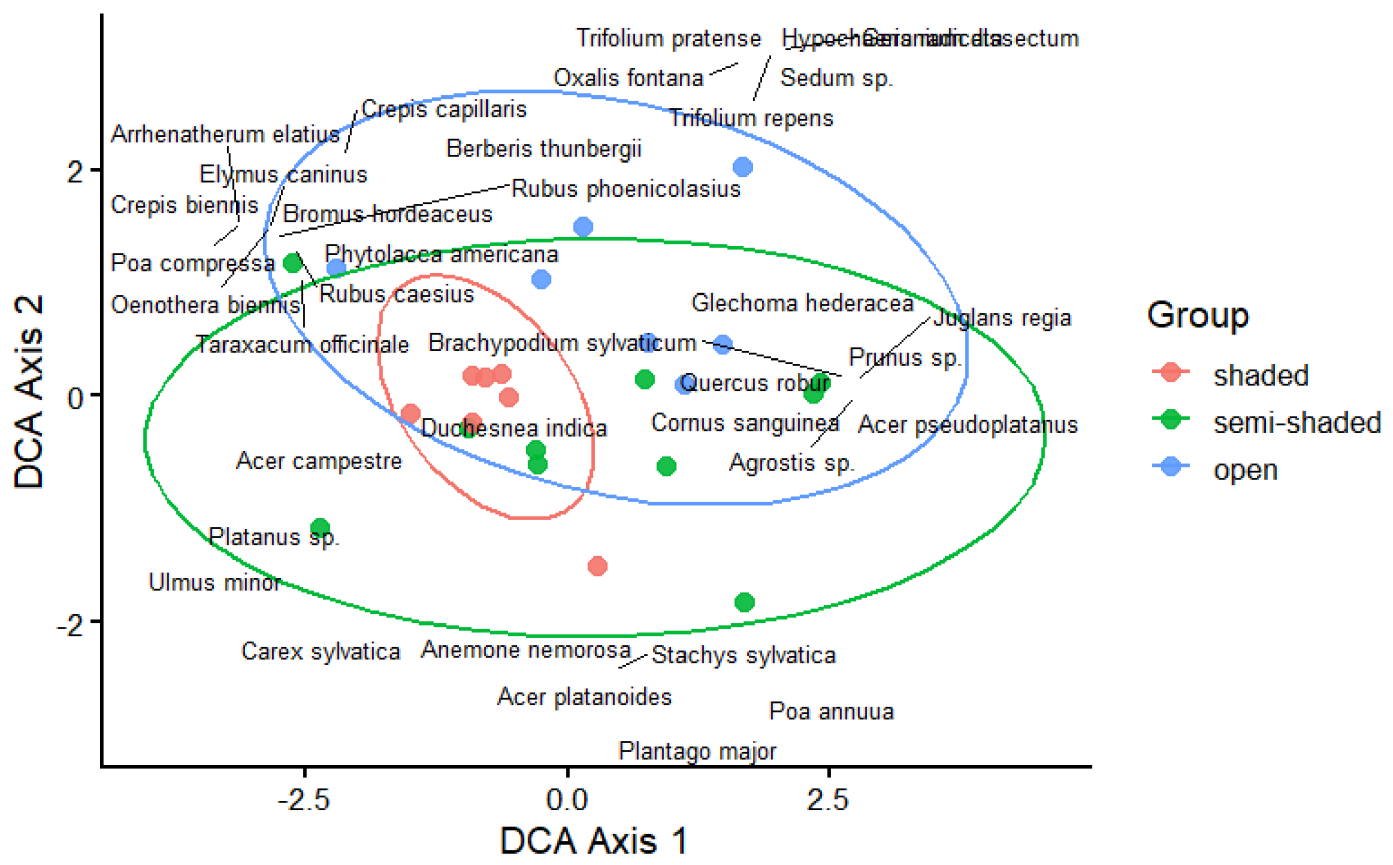
Detrended Correspondence Analysis (DCA) ordination of vegetation plots associated with *Duchesnea indica*. Points represent individual plots and are coloured according to habitat group (open, semi-shaded, and shaded). Ellipses indicate the 95% confidence intervals around the centroid of each habitat group. Species labels show selected plant taxa contributing most strongly to the variation in species composition. The first DCA axis represents the primary gradient of species turnover among the sampled habitats.

**Figure 6:**
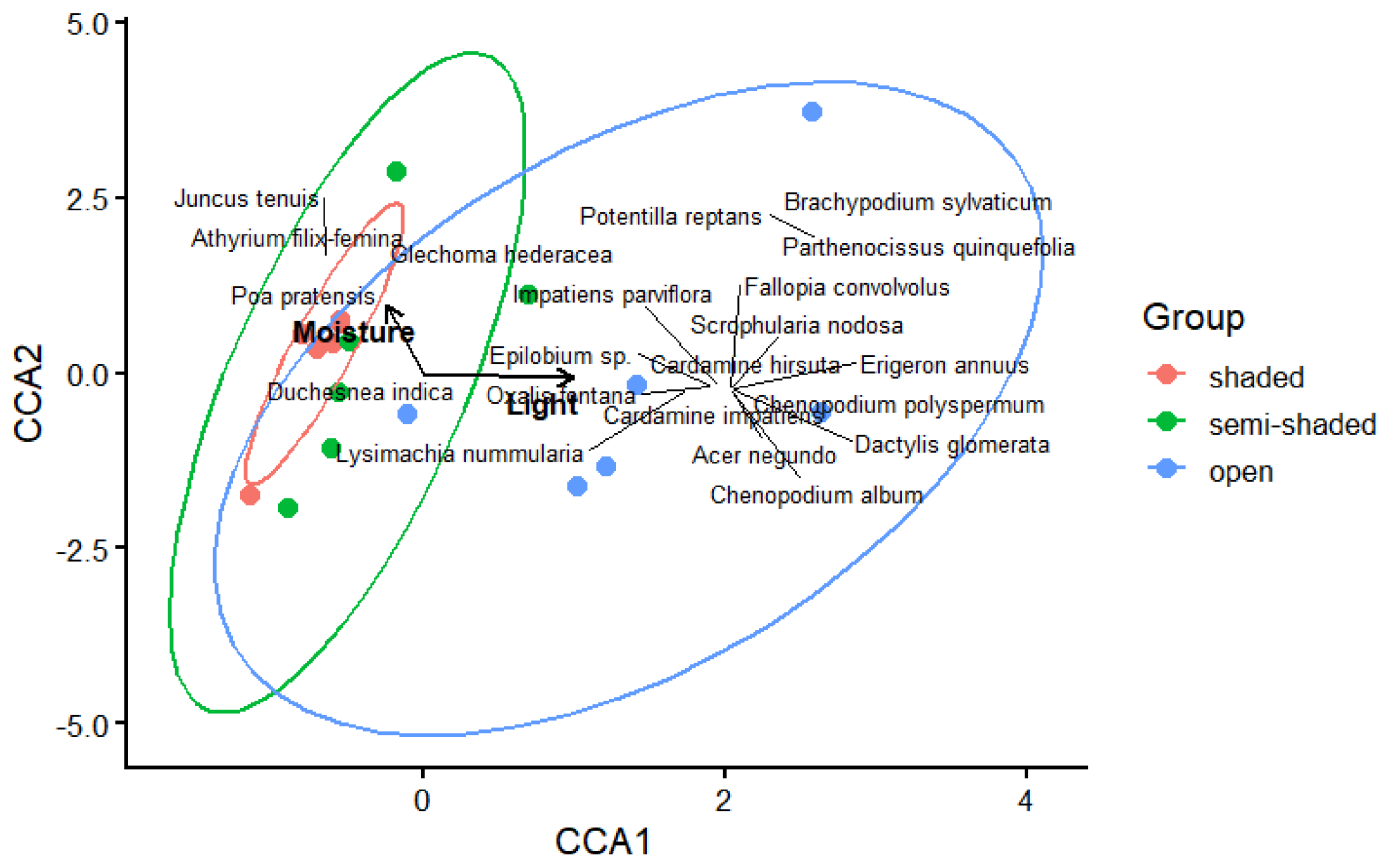
Canonical Correspondence Analysis (CCA) ordination of vegetation plots associated with *Duchesnea indica*, constrained by ecological indicator values for light and moisture. Points represent individual plots coloured according to habitat group (open, semi-shaded, and shaded), and ellipses indicate the 95% confidence intervals around group centroids. Arrows represent the direction and strength of the environmental gradients. To improve the readability of the ordination diagram, only the 20 most representative species and *D. indica* were displayed.

**Table 1:** Characteristics of contrasting habitats occupied by *D. indica* in its introduced range (DI1–DI24, *n* = 24): mean plant species richness, vegetation cover, and *D. indica* cover (±standard deviation).

	Shaded		Semi-Shaded		Open Habitat	
	Mean	SD	Mean	SD	Mean	SD
Plant species richness	13.9	4.6	21.6	8.1	20.9	9.8
Vegetation cover (%)	79.3 ^+^	11.3	74 ^+^	13.3	72.9	22.0
*D. indica* cover (%)	56.1	33.5	29.5	27.1	21.4	11.0

^+^ Herb layer cover.

**Table 2:** Mean, minimum, and maximum values of measured soil properties in habitats occupied by *Duchesnea indica*. Differences among habitat groups were tested using one-way ANOVA (R version 4.5.2). Asterisks (*) indicate statistically significant differences among habitat groups (*p* < 0.05).

Soil Variable	Mean	Min	Max
pH (1:5 KCl)	6.91	4.57	8.10
K_2_O (mg/kg)	260.07	62.27	549.45
P_2_O_5_ (mg/kg)	194.90	74.16	289.64
N (mg/kg)	2996.53	679.59	5833.64
C (mg/kg)	49,663.58	12,099.69	84,762.75
S (mg/kg)	728.31	107.43	1225.31
Cr (mg/kg)	97.06	61.00	162.00
Mn (mg/kg)	790.92	488.50	1057.00
Fe (mg/kg)	36,413.50	26,751.50	44,163.00
Ni (mg/kg)	40.47	18.00	55.00
Cu (mg/kg)	43.69 *	26.50	67.00
Zn (mg/kg)	153.53	58.50	215.50
As (mg/kg)	16.14	7.50	25.50
Pb (mg/kg)	37.17	13.50	70.50

## Data Availability

The data presented in this study are available from the corresponding author upon reasonable request.

## Appendix A

**Table A1:** List of vegetation plots (relevés) containing *Duchesnea indica*. For each plot, the sampling location (including geographic coordinates), sampling date, habitat, plot size, *D. indica* cover, and total vegetation cover are provided. In forest (shaded) and forest-edge (semi-shaded) habitats, total vegetation cover refers to the herb layer.

Relevé	Sampling Location	Coordinate x	Coordinate y	Sampling Date	Habitat	Plot Size (m^2^)	Cover of Duchesnea (%)	Vegetation Cover (%)
DI1	Brezje	46.5402778	15.6916147	3. 6. 2020	shaded	60	87.5	90
DI2	Tezno	46.5425915	15.6903699	3. 6. 2020	semi-shaded	13	5	80
DI3	Zrkovci	46.5398739	15.6914310	4. 6. 2020	open	5	37.5	100
DI4	Zrkovci	46.5385788	15.6989566	4. 6. 2020	shaded	6	15	75
DI5	Makedonska ul.	46.5438458	15.6578545	11. 6. 2020	open	6	15	80
DI6	Muratova ul.	46.5450020	15.6599925	11. 6. 2020	open	20	15	40
DI7	Jamova ul.	46.5458109	15.6596817	11. 6. 2020	open	20	37.5	95
DI8	Miklavž	46.5143605	15.6893506	11. 6. 2020	semi-shaded	20	37.5	80
DI9	Miklavž	46.5148531	15.6904222	11. 6. 2020	open	7	15	50
DI10	Miklavž	46.5112288	15.6880769	11. 6. 2020	semi-shaded	20	87.5	90
DI11	Rogoza	46.5049377	15.6855758	16. 6. 2020	semi-shaded	25	15	70
DI12	Betnavski gozd	46.5348664	15.6380024	16. 6. 2020	shaded	25	37.5	70
DI13	Ljubljanska ul.	46.5355565	15.6366184	16. 6. 2020	shaded	10	62.5	85
DI14	Betnavska c.	46.5379918	15.6369936	16. 6. 2020	semi-shaded	6	15	75
DI15	Ertlova ulica	46.5381881	15.6374848	16. 6. 2020	shaded	6	87.5	90
DI16	Tabor	46.5375200	15.6377902	16. 6. 2020	open	14	15	75
DI17	Betnava	46.5291634	15.6420827	16. 6. 2020	shaded	4	87.5	85
DI18	Betnava	46.5293401	15.6418571	16. 6. 2020	semi-shaded	15	62.5	70
DI19	Streliška c.	46.5290248	15.6404379	16. 6. 2020	semi-shaded	20	37.5	60
DI20	Park Betnava	46.5298201	15.6403098	16. 6. 2020	shaded	6	15	60
DI21	Kosovelova ul.	46.5447470	15.6647038	7. 7. 2020	open	30	15	70
DI22	C. XIV. divizije	46.5420092	15.6798831	7. 7. 2020	semi-shaded	25	15	50
DI23	V borovju	46.5292072	15.6613138	7. 7. 2020	semi-shaded	32	5	70
DI24	Lahova ul.	46.5297645	15.6623867	7. 7. 2020	semi-shaded	20	15	95
